# The hydrophilic extract from a new tomato genotype (named DHO) kills cancer cell lines through the modulation of the DNA damage response induced by Campthotecin treatment

**DOI:** 10.3389/fonc.2023.1117262

**Published:** 2023-06-20

**Authors:** Daniela Barone, Carmelina Antonella Iannuzzi, Iris Maria Forte, Maria Carmen Ragosta, Maria Cuomo, Milena Dell’Aquila, Angela Altieri, Antonella Caporaso, Rosa Camerlingo, Maria Manuela Rigano, Daria Maria Monti, Amalia Barone, Paola Imbimbo, Luigi Frusciante, Marcellino Monda, Margherita D’Angelo, Michelino De Laurentiis, Antonio Giordano, Luigi Alfano

**Affiliations:** ^1^ Cell Biology and Biotherapy Unit, Istituto Nazionale Tumori-Istituto di Ricovero e Cura a Carattere Scientifico (IRCCS)-Fondazione G. Pascale, Napoli, Italy; ^2^ Department of Medicine, Surgery and Neuroscience, University of Siena and Istituto Toscana Tumori (ITT), Siena, Italy; ^3^ Department of Agricultural Sciences, University of Naples Federico II, Naples, Italy; ^4^ Department of Chemical Sciences, University of Naples Federico II, Naples, Italy; ^5^ Unit of Dietetics and Sports Medicine, Department of Experimental Medicine, Section of Human Physiology, Università degli Studi della Campania “Luigi Vanvitelli”, Naples, Italy; ^6^ Department of Breast and Thoracic Oncology, Istituto Nazionale Tumori-IRCCS-Fondazione G. Pascale, Napoli, Italy; ^7^ Sbarro Institute for Cancer Research and Molecular Medicine, Center for Biotechnology, College of Science and Technology, Temple University, Philadelphia, PA, United States

**Keywords:** DNA damage response, *Solanum lycopersicum*, homologous recombination, DNA repair, single strand annealing

## Abstract

**Introduction:**

DNA double-strand breaks are the most toxic lesions repaired through the non-homologous and joining (NHEJ) or the homologous recombination (HR), which is dependent on the generation of single-strand tails, by the DNA end resection mechanism. The resolution of the HR intermediates leads to error-free repair (Gene Conversion) or the mutagenic pathways (Single Strand Annealing and Alternative End-Joining); the regulation of processes leading to the resolution of the HR intermediates is not fully understood.

**Methods:**

Here, we used a hydrophilic extract of a new tomato genotype (named DHO) in order to modulate the Camptothecin (CPT) DNA damage response.

**Results:**

We demonstrated increased phosphorylation of Replication Protein A 32 Serine 4/8 (RPA32 S4/8) protein in HeLa cells treated with the CPT in combination with DHO extract with respect to CPT alone. Moreover, we pointed out a change in HR intermediates resolution from Gene Conversion to Single Strand Annealing through the modified DNA repair protein RAD52 homolog (RAD52), DNA excision repair protein ERCC-1 (ERCC1) chromatin loading in response to DHO extract, and CPT co-treatment, with respect to the vehicle. Finally, we showed an increased sensitivity of HeLa cell lines to DHO extract and CPT co-treatment suggesting a possible mechanism for increasing the efficiency of cancer therapy.

**Discussion:**

We described the potential role of DHO extract in the modulation of DNA repair, in response to Camptothecin treatment (CPT), favoring an increased sensitivity of HeLa cell lines to topoisomerase inhibitor therapy.

## 1 Introduction

Genome instability is one of the leading causes of cancer development (1); various sources of DNA damage, both endogenous and exogenous, can affect the double helix structure. The most toxic lesions of DNA are double strand breaks (DSBs), which are repaired by two principal mechanisms, NHEJ or HR, to preserve genome stability. NHEJ is an error prone mechanism generating high number of insertions or deletions to repair the damaged DNA (2) working through all the cell cycle phases; conversely, the HR is restricted to the S/G2, given its dependency on the presence of the sister chromatid for the correct DNA repair (3). The starting point of the HR is the DNA end-resection mechanism necessary for the generation of the 3’ long single strand DNA tails, which are bounded by the RPA complex to prevent helical torsional stress and favoring the resolution of the HR intermediates through the Gene Conversion (GC), Single Strand Annealing (SSA) or alternative End-joining (alt-EJ) (4, 5). In particular, the GC process involves the DNA repair protein RAD51 homolog 1 (RAD51) protein for the stand exchange leading to the error free repair (6); the SSA requires the binding of RAD52 protein to the exposed repeats flanking the DBSs, which favor the annealing (7); finally, the alt-EJ is regulated by the Poly [ADP-ribose] polymerase 1 (PARP1) protein mediating the annealing of microhomologies for the proper DNA repair (8). Many HR proteins are described to be mutated in cancer samples as: Breast cancer type 1 susceptibility protein (BRCA1) in breast cancer (9), DNA repair protein RAD51 homolog 2 (RAD51B) in uterine leiomyoma (10) or ATP-dependent DNA helicase Q4 (RECQL4) in basal and squamous cell skin carcinomas (11), pointing out the relevance of the HR in the tumor development. More recently, Ashworth and colleagues described the synthetic lethal phenomenon as a marked sensitivity of the BRCA1 -/- cell lines to the PARP-1 inhibitors, opening a new era for cancer therapy (12). Given the high number of resistance mechanisms, many screenings to PARP1 inhibitors were performed in order to identify new lethal interactions as a possible druggable mechanism (13). Many drugs used in cancer therapy are made from the natural compounds as Taxol, from bark of the Pacific Yew tree (14), or Camptothecin, from the Camptotheca Acuminata (15). Recently, the National Cancer Institute developed a research program, called the NCI Program for Natural Products Discovery (NPNPD), in order to identify novel natural compounds for cancer therapy evidencing the interest of the research community in the new molecules identification from natural compounds for tumor treatment. Petruk et al. described the potential protective role of the hydrophilic extract of a new tomato genotype (named DHO) onto the UVA induced damage, probably through the action of the antioxidant Vitamin C (16). Given that the UV rays induce the activation of the Intra-S-phase checkpoint, which is essential to prevent mitosis in damaged cells (17), we checked the possible involvement of this tomato extract in the modulation of the DNA damage response (DDR) followed by Camptothecin treatment. Here we demonstrated the effects of the DHO hydrophilic extract on the RPA32 S4/8 phosphorylation induced by CPT leading an altered RAD52 chromatin loading; moreover, the use of the DHO extract in combination with the CPT treatment induces a marked sensitivity in HeLa cell lines to topoisomerase inhibitor treatment showing a promising mechanism for cancer therapy.

## 2 Material and methods

### 2.1 Tomato extracts

DHO is a tomato genotype previously obtained at the laboratory of the Department of Agricultural Science (University of Naples Federico II). The Hydrophilic extracts from the DHO tomato line were obtained according to the procedure reported by Petruk et al. (16) 2 g of DHO frozen powder were solubilized in 25 mL of 70% methanol and the extraction was done into an ultrasonic bath (Branson 5200 Ultrasonic Corp.) for 60 min at 30°C. The sample was dried by rotovapor (R-210, Buchi), and re-dissolved in 1 ml of DMSO 5% in PBS (1 mL).

### 2.2 Cell cultures

Cervix adenocarcinoma (HeLa) cells were purchased from ATCC and cultured as recommended. To silence 53BP1 protein, we used a sequence GAAGGACGGAGUACUAAUAdTdT (18) transfected with the Dharmafect 1 reagent. A commercial non-targeting-siRNA (siCTR) was used as a negative control (Horizon Discovery).

### 2.3 Antibodies and western blot

The following antibodies were used: RPA32 (A300-244A, Bethyl Laboratories), RPA32 S4/S8 (A300-245A, Bethyl Laboratories), Lamin A/C (#4777, Cell Signalling), RAD52 (sc-365341, Santa Cruz Biotechnology), RAD51 (sc-8349, Santa Cruz Biotechnology), ERCC1 (NB500-704, Novus Biological), RAD52 (sc-365341, Santa Cruz Biotechnology), PARP1 (sc-8007, Santa Cruz Biotechnology). For total protein extraction, cells were lysed at 4°C in 50 mM HEPES pH7.5, 1% Triton X-100, 150 mM NaCl, 5 mM EGTA, supplemented with protease and phosphatase inhibitor cocktail (Roche Applied Science). Lysates were clarified by centrifugation at 10.000 × g for 20 min. Lysates containing equal amounts of proteins, estimated through the Bradford assay (Bio- Rad), were subjected to SDS-page. The chemiluminescent images were obtained using the ImageQuant LAS 500 (GE Healthcare). Band densitometry values inserted in all western blot figures indicate the levels of all phosho-proteins normalized to correspondent total protein and the last are normalized with the internal reference protein. 

### 2.4 Immunofluorescence

HeLa cells, grown on glass coverslips, were fixed with 4% paraformaldehyde and permeabilized with 0.2% Triton X-100. Samples were blocked 10 min in 1% BSA at RT and incubated 1 hour with anti-pRPA32 S4/8 (1:200, A300-245A, Bethyl Laboratories), anti-γH2AX (1:300, ab20669, Abcam), at 37°C. After washing, samples were incubated 45 min at 37°C with AlexaFluor 594-conjugated chicken anti-rabbit (Thermo Fischer Scientific) and analysed with a Zeiss LSM900 confocal microscope. The foci intensity and foci per cell values were calculated by Fiji software

### 2.5 Cell fractionation

Cell fractionation was performed as previously described by Ishii et al. with minor modifications (19). Briefly, 3 × 10^6^ cells, per condition, were collected and resuspended in 200 μl of CSK buffer (10 mM PIPES pH 6.8, 100 mM NaCl, 300 mM MgCl2, 1 mM EGTA, 1 mM DTT, 0.1% Triton X- 100, 0.34 M sucrose) supplemented with protease and phosphatase inhibitors and kept 5 min on ice. The soluble cytoplasmic fraction (S) was separated from nuclei (P) by 4 min centrifugation at 1300 × g at 4°C. The P fraction was washed with CSK then resuspended in 200 μl of ‘western blot buffer’, sonicated and centrifuged for 30 min at 4°C at 10.000 × g. Following SDS-PAGE, samples were analyzed by western blot with the indicated antibodies.

### 2.6 Cell cycle profile

For DNA content analysis, HeLa cells were fixed in ice-cold 70% ethanol at –20°C following staining with 5 μg/ml propidium iodide and 0.25 mg/ml RNaseI treatment (Sigma Aldrich) in PBS1x. At least 10.000 cells/condition were analysed by FACS Canto (Becton Dickinson, San Jose, CA, USA). Data were analyzed through the CellQuest Software (Becton Dickinson) (20).

### 2.7 DNA damage mechanisms reporter assay

To generate HeLa cells stable expressing the reporter plasmid pDR-GFP (pimEJ5GFP or hprtSAGFP), cells were transfected with the pDR-GFP plasmid (21) (gift from Maria Jasin, Addgene plasmid #26475; http://n2t.net/addgene:26475; RRID : Addgene_26475), pimEJ5GFP plasmid (7) (gift from Jeremy Stark, Addgene plasmid #44026; http://n2t.net/addgene:44026; RRID : Addgene_44026) or hprtSAGFP plasmid (22) (gift from Maria Jasin, Addgene plasmid #41594; http://n2t.net/addgene:41594; RRID : Addgene_41594) and selected with puromycin. HeLa pDRGFP (pimEJ5GFP or hprtSAGFP) cell lines were co-transfected with the coding plasmid for the endonuclease I-SceI (21) (pCBASceI, a gift from Maria Jasin, Addgene plasmid #26477; http://n2t.net/addgene:26477; RRID : Addgene_26477). Upon 48 hours of incubation, we analyzed the GFP values (as a HR, NHEJ or SSA frequencies) through the FACS ARIA III (BD). Data were analyzed through Diva 8.0 software.

### 2.8 MTS

CPT and DHO were dissolved in DMSO as a stock and then diluted in culture medium. The cells were seeded in 96-well plates 24 h prior to treatment with increasing concentrations (15.6-10000 nM) of CPT or increasing concentrations (0.25-3 mg/ml) of DHO. As a control, cells were treated with the maximum amount of DMSO used to deliver the compounds. At 72 h after treatment, cell viability was evaluated by MTS assay (CellTiter 96^®^ AQueous One Solution Cell Proliferation Assay; Cat. no. G3582; Promega, Milan, Italy), following the manufacturer’s instructions. The half maximal inhibitory concentration (IC_50_) values were calculated using GraphPad Prism Software, version 5.01 for Windows. DMSO exhibited no toxic effect on any of the cell lines (data not shown).

### 2.9 Clonogenic assay

300 cells were seeded in each well of 24-wells plates and either treated with the indicated doses of CPT or not treated. Cells were incubated for 10 days. Colonies were counted after fixation with methanol and staining with crystal violet.

### 2.10 Random plasmid integration assay

Random Plasmid Integration Assay was performed as previously described with minor modifications (21). HeLa cells were transfected with 2 μg of pCMV-HIS (CV003, Sino Biological) (per 6cm dish) linearized (by ApaI restriction enzyme digestion) and 1 μg of pEGFP-C1 with Lipofectamine 2000 transfection reagent according to the manufacturer’s instructions. Six hours after plasmid transfection, cells were seeded into three 6 cm dishes at 5x104 cells and treated or not with 1 mg/ml DHO hydrophilic extract; moreover, we plated an additional 6 cm dish at 30x104 cell density for the GFP cytofluorimetric analysis after 24 hours. Forty-eight hours after re-seeding, plates were cultured in presence of 0.8 mg/ml of hygromycin (H3274, Sigma Aldrich) for 15 days and subsequently stained with 2% (w/v) crystal violet solution. Plasmid integration efficiency was analyzed as the percentage of hygromycin-resistant cells normalized to the transfection efficiency.

### 2.11 Drug combination studies

For drug combination studies, we first determined the 72-h IC50 values of CPT through MTS assay in the HELA cells 1000/well. Subsequently, based on the CPT IC50 values, we challenged the HELA cells for 72 h with CPT and DHO, both alone and in combination at various concentrations non-inconstant ratio (we used 1mg/ml of DHO for all combinations) and assessed cell viability through MTS assay. Synergism, additivity, or antagonism were determined by calculating the combination index (CI) according to the Chou-Talalay equation, using CalcuSyn software 1.1.1 (BioSoft, Cambridge, UK). CI <1 indicates synergism, CI = 1 additive effect, and CI >1 antagonism. 

### 2.12 Statistical analysis and reproducibility

Paired two-sided Student’s t-test was used to compare the means of two matched groups; p<0.05 was considered statistically significant. Representative experiments are shown out of three independent ones; detailed information on P-values is listed in the individual figure legends.

## 3 Results

### 3.1 Tomato hydrophilic extract increases phosphorylation of the RPA32 protein in response to CPT

**Figure 1:**
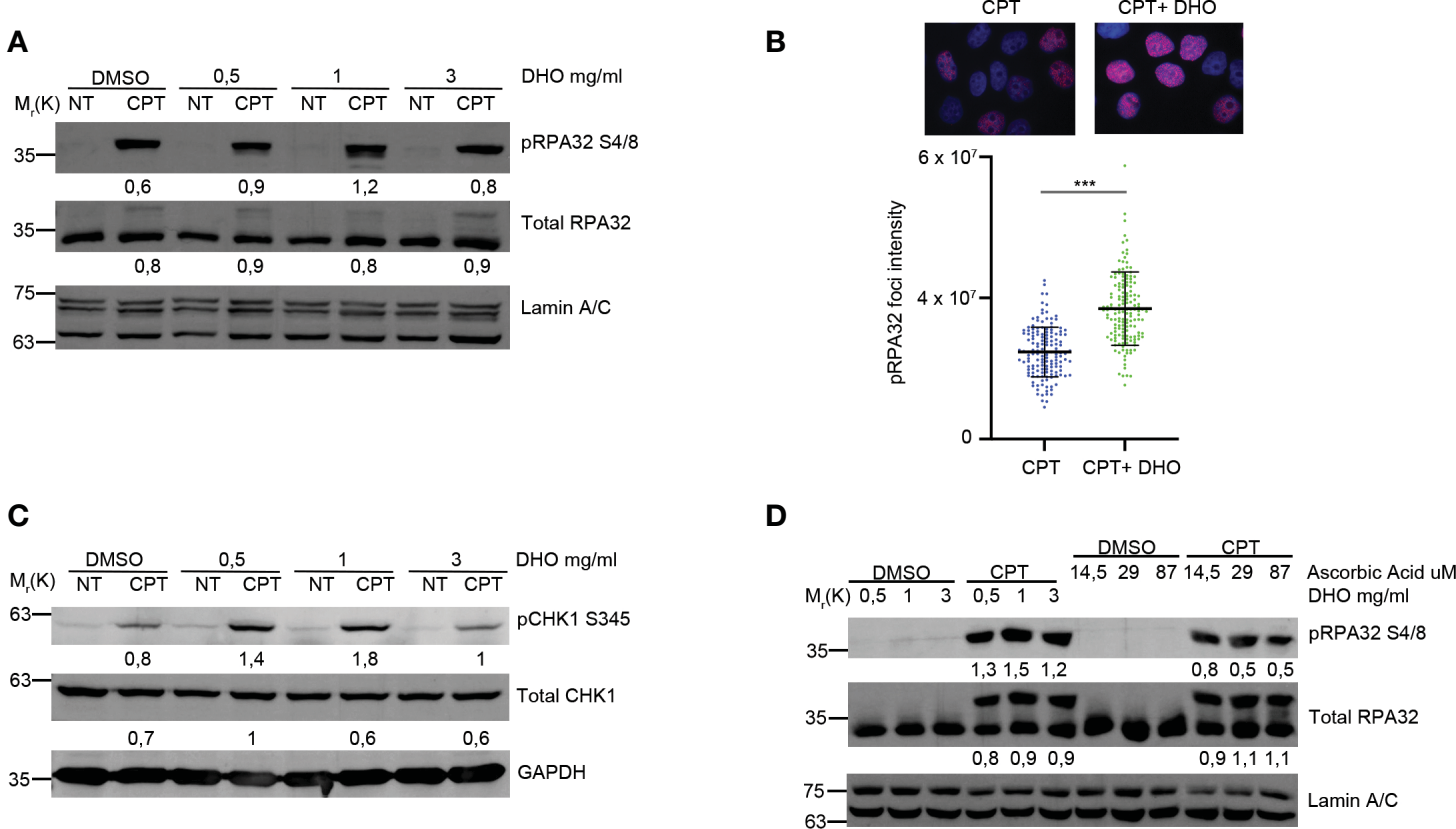
DHO hydrophilic extract increases the RPA32 S4/8 phosphorylation in response to CPT respect to CPT alone. **(A)** Western blot analysis of HeLa cells pre-treated, for one hour, with different concentrations (0.5, 1, 3 mg/ml) of DHO extract and DMSO followed by a treatment with 1 μM CPT for additional two hours. Total RPA32 and Lamin A/C are loading as control of pRPA32 S4/8 and total RPA32, respective. The values obtained are reported in the blot images. **(B)** Immunofluorescence analysis of pRPA32 S4/8 foci intensity in HeLa cells pretreated for one hour with DHO 1mg/ml or DMSO, followed incubation by 1 μM CPT for additional two hours. The histogram reports three independent experiments with standard deviations (SD). Statistically significant differences are indicated with: ***significant (P < 0.001). **(C)** Western blot analysis of HeLa cells treat with DHO with different concentrations (0.5, 1, 3 mg/ml) and CTP (1 µM) alone and in combination. CHK1 S345 phosphorylation (pCHK1 S345). Total CHK1 and GAPDH are loading as control of pCHK1 S345 and total CHK1, respective. The values obtained are reported in the blot images. **(D)** Western blot analysis of HeLa cells pre-treated for one hour with different DHO (0.5, 1, 3 mg/ml) or Ascorbic Acid (14.5, 29 and 87 μM) concentrations followed by two hours of CPT treatment. pRPA32 S4/8 was normalized as indicated above.

Petruk et al. described a potential role of the DHO extract in the protection against UV rays (16); given the effect of the UV rays onto the activation of the HR (23), we checked the possible effects of the DHO hydrophilic extract onto the regulation of the HR in response to the CPT treatment, notoriously involved in the DSBs formation activating DNA end-resection mechanism for the proper HR (23). First of all, we pre-treated HeLa cells with different concentration of DHO, for one hour, followed by CPT treatment for additional two hours. As reported in **Figure 1A**, CPT induces the RPA32 S4/8 phosphorylation but the co-treatment with 1 mg/ml of DHO increases the activation of RPA32. Moreover, the use of 3mg/ml of DHO extract induces a pRPA S4/8 protein signal reduction given the presence of different compounds in the hydrophilic fraction. Analysis of phosho-RPA32 S4/8 foci, through immunofluorescence, reveals an increased signal intensity in HeLa cells treated with a DHO/CPT combination respect to the CPT alone (**Figure 1B**). CPT induced ATR checkpoint activation leading to the CHK1 phosphorylation at S345 (pCHK1 S345) (24); we demonstrated the increased CHK1 activation in response to CPT, in combination with DHO extract, respect to Topo I inhibitor alone (**Figure 1C**). As previously reported, UVA rays protection mediated by the DHO extract depends probably on the high concentration of ascorbic acid (AsA) present in this tomato extract (16). Therefore, we checked the possible role of Vitamin C in RPA32 phosphorylation modulation through the HeLa cells pre-treatment with different concentration of AsA, which were the amounts present in DHO (24), followed by CPT treatment for additional two hours. As showed in **Figure 1D**, AsA was not able to increase the RPA32 phosphorylation respect to the DHO extract.

### 3.2 DHO treatment does not alter the cell cycle profile and γH2AX signal in HeLa cells

**Figure 2:**
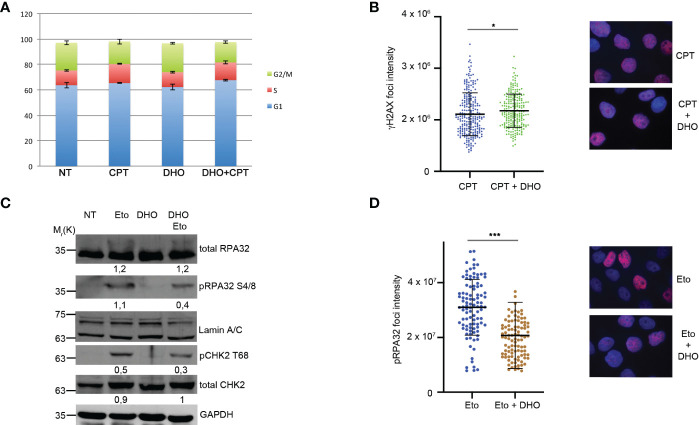
DHO hydrophilic extract do not alter cell cycle distribution anh H2AX activation **(A)** Cell cycle profile of HeLa cells pre-treated with 1 mg/ml of DHO or vehicle (DMSO) for one hours followed by incubation with CPT (1 µM) for additional two hours, was analysed through flow cytometry upon propidium iodide (PI) staining. **(B)** Immunofluorescence analysis of γH2AX foci intensity in HeLa cells pretreated for one hour with DHO 1mg/ml or vehicle (DMSO), followed incubation by 1 μM CPT for additional two hours. The histogram reports the mean of three independent experiments with standard deviations (SD). Statistically significant differences are indicated with: *significant (P < 0.05). **(C)** Western blot analysis of HeLa cells pre-treated for one hour with DHO at 1 mg/ml or vehicle (DMSO), followed by two hours of Etoposide (20 μM) incubation. RPA S4/8 and pCHK1 S345 or vehicle (DMSO), followed by two hours of Etoposide (20 μM) incubation. RPA S4/8 and pCHK1 S345 were normalized to those of each respective total RPA32 and CHK1 controls. **(D)** Immunofluorescence analysis of pRPA32 S4/8 foci intensity in HeLa cells pre-treated with 1mg/ml of DHO followed by incubation with Etoposide 20 µM for additional two hours. The histogram reports the mean of three independent experiments with standard deviations (SD). Statistically significant differences are indicated with: ***significant (P < 0.001).

RPA32Ser 4/8 protein was phosphorylated in response to DNA damage as a marker of DNA end resection (25) as a part of S-phase dependent HR. In order to demonstrate that the RPA32 phosphorylation increase, treated with DHO in combination with CPT, was not dependent by an increased S-phase, we performed a cell cycle analysis showing a same HeLa cells distribution through cell cycle phases suggesting a possible role of DHO in DNA repair (**Figure 2A**). Moreover, we analyzed the possible involvement of DHO in DNA damage induction measured through γH2AX foci; as reported in **Figure 2B**, the presence of hydrophilic extract induced a slighly increase of the protein signal intensity of HeLa cells treated with CPT and DHO respect to the topoisomerase inhibitor I alone. Moreover, we checked the effect of DHO onto the DNA damage response induced by Etoposide, a Topoisomerase II (Topo II) inhibitor, showing a reduced activation of pRPA32 S4/8 and pCHK2 T68 response in combination with the Etoposide respect to the Topo II inhibitor alone (**Figure 2C**). Consistently with the previous experiment, the analysis of the pRPA 32 S4/8 foci intensity confirmed a reduced protein levels in HeLa cells treated with the combination between the CPT and DHO respect to the CPT alone (**Figure 2D**).

### 3.3 Tomato hydrophilic extract treatment does not modify the HR and NHEJ activity

**Figure 3:**
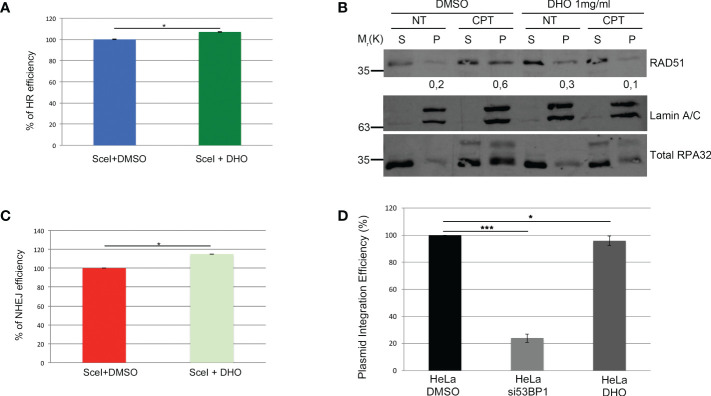
DHO hydrophilic extract impairs RAD51 chromatin loading **(A)** HeLa DR-GFP cells were transfected with the coding plasmid for the *SceI* restriction enzyme in presence of 1 mg/ml of DHO or vehicle (DMSO) and incubated for 48 hours. FACS analysis measurement of GFP levels was used to calculate %HR frequency compared with DMSO alone which was set as 100% (control). Data represent the mean % ± SD obtained from three independent experiments. Statistically significant differences are indicated with: *significant (P < 0.05). **(B)** Chromatin enhriched purification of HeLa cells pretreated or not with the DHO extract at 1 mg/ml followed by 1 μM CPT treatment for additional two hours. Cells were then lysed to obtain a soluble (S) and a chromatin-enriched (P, as pellet) fraction. Western blotting of RAD51 was performed to analyse the loading onto chromatin of the indicated proteins, involved in the cell response to DNA damage. Total RPA32 and Lamin A/C were used as controls of the supernatant or the chromatin-enriched fraction, respectively. **(C)** HeLa pimEJ5-GFP cells were transfected with the plasmid encoding the *SceI* restriction enzyme followed by incubation with 1 mg/ml of DHO extract or vehicle (DMSO) for 48 hours followed by FACS analysis measurement of GFP levels to calculate %NHEJ frequency compared with control cells which were set as 100%. Data represent the mean ± SD obtained from three independent experiments. Statistically significant differences are indicated with: *significant (P < 0.05). **(D)** HeLa cells were transfected with the pCMV-His empty vector linearized with the *ApaI* restriction enzyme and pEGFP-C1 in presence of siCTR or si53BP1 followed by incubation with the vehicle (DMSO) or 1 mg/ml DHO hydrophilic extract for 15 days and stained with 2% (w/v) crystal violet solution. Data represent the mean % ± SD obtained from three independent experiments. Statistically significant differences are indicated with: *significant (P < 0.05), ***significant (P < 0.001).

**Figure 4:**
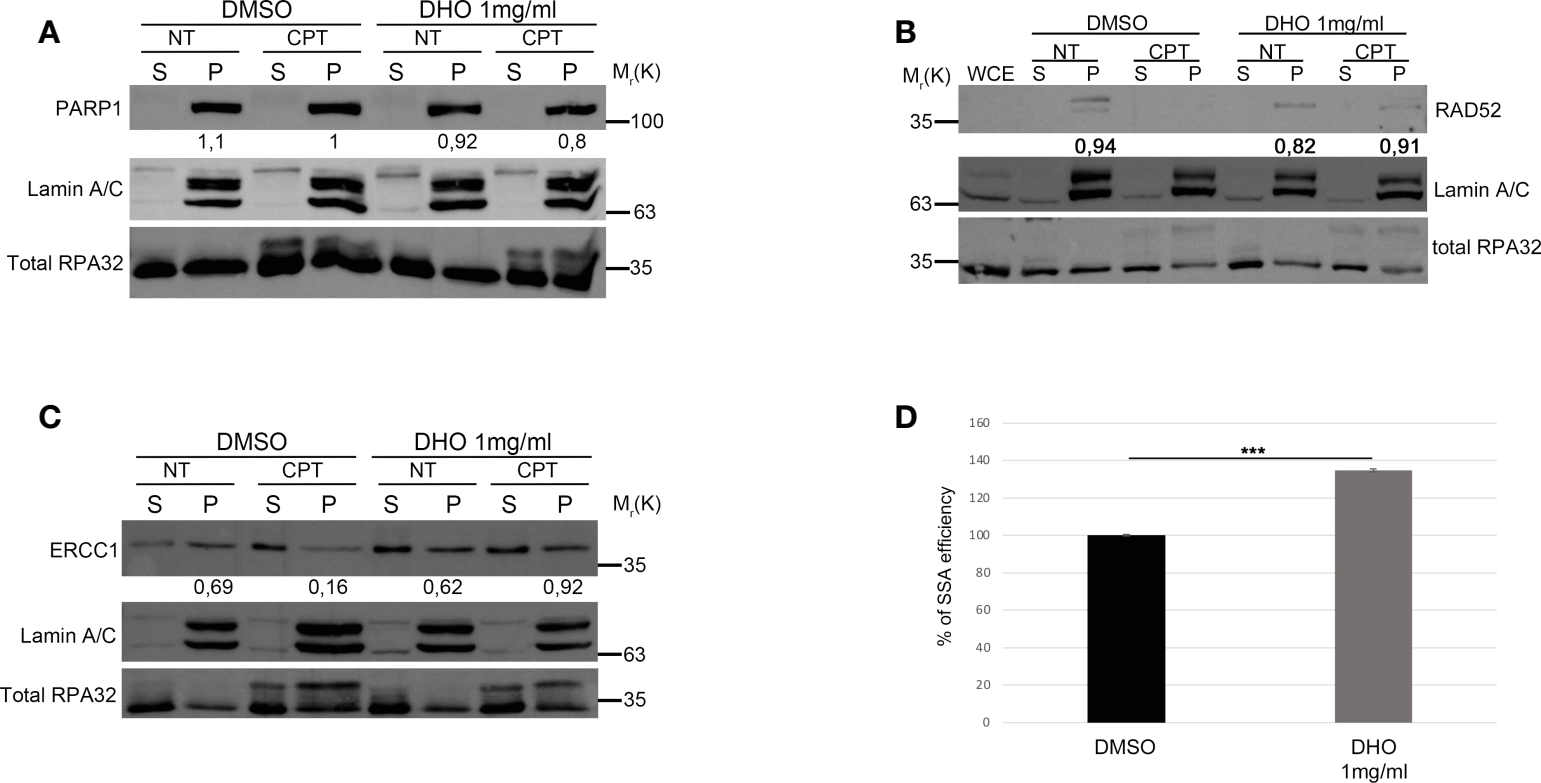
DHO hydrophilic extract increases the Single Strand Annealing activity in response to CPT treatment **(A)** Chromatin enhriched purification of HeLa cells pretreated or not with the DHO extract at 1 mg/ml followed by 1 μM CPT treatment for additional two hours. Cells were then lysed to obtain a soluble (S) and a chromatin-enriched (P, as pellet) fraction. Western blotting was performed to analyse the loading onto chromatin of the Poly (ADP-ribose) polymerase (PARP1) protein, involved in the cell response to DNA damage. Total RPA32 and Lamin A/C were used as controls of the supernatant or the chromatin-enriched fraction, respectively. **(B)** Chromatin enhriched purification of HeLa cells performed as previously for the analysis of RAD52 chromatin loading. **(C)** Chromatin enhiched purification of HeLa cells was performed as previously described followed by incubation with ERCC1 antibody. **(D)** HeLa hprtSAGFP cells were transfected with the plasmid encoding the *SceI* restriction enzyme followed by incubation with 1 mg/ml of DHO extract or vehicle (DMSO) for 48 hours followed by FACS analysis measurement of GFP levels to calculate %SSA frequency compared with control cells which were set as 100%. Data represent the mean % ± SD. obtained from three independent experiments. Statistically significant differences are indicated with: ***significant (P < 0.001).

The two main pathways involved in the DSBs repair were the HR and NHEJ; the DNA repair pathway choice, between the HR and NHEJ, is one of the most studied mechanisms but it is poorly understood (26). The resolution of the HR depends by the DNA end-resection, important for the generation of the single strand 3’ tails (27). In order to check the possible involvement of the DHO extract in the HR regulation, we used the HeLa cells stably expressing the DR-GFP (HeLa DR-GPF) reporter plasmid to measure the Gene Conversion (GC) (21). We transfected HeLa DR-GFP with the coding plasmid for the endonuclease I-SceI, necessary for the generation of DSBs, and incubated in presence or not of DHO extract for 48 hours. As reported in **Figure 3A**, the presence of DHO does not alter the GC frequency. Consistently, HeLa chromatin-enriched purification showed a marked reduction of RAD51 protein loading, a marker of homologous stand exchange (28), in the presence of DHO confirming an impairment of GC frequency (**Figure 3B**). Recently, Löbrich et al. described the possible involvement of the end-resection in the resolution of the NHEJ (29). To check if the DHO extract could be involved in the DSBs repair *via* NHEJ we used the HeLa cells stably expressing the pimEJ5-GFP (7), a reporter system for the NHEJ, transfected with the coding plasmid for the endonuclease I-SceI and incubated in presence or not of 1 mg/ml of DHO extract for 48 hours. As reported in **Figure 3C**, DHO treatment does not affect the NHEJ activity; moreover, through a random integration assay we confirmed the unaffected NHEJ efficiency in presence of DHO extract (**Figure 3D**). The resected DNA upon DNA damage, could be repaired by three pathways: GC, alternative end-joining (alt-EJ) and Single Strand Annealing (SSA). Given that DHO extract does not affect the GC frequency, we checked the possible effect of DHO onto the chromatin loading of PARP1, a master regulator of alt-EJ (30); as reported in **Figure 4A**, DHO induced a decrease of PARP1 protein onto chromatin fraction respect to CPT alone. Finally, we analysed the involvement of SSA mechanism in the resolution of the DSBs through the analysis of RAD52 chromatin loading. The CPT treatment induces a dissociation of RAD52 from the chromatin fraction (P) (**Figure 4B**) favoring the loading of the RAD51 (**Figure 3B**), consistent with the DNA repair through the GC. Surprisingly, the co-treatment of the DHO extract with the CPT induces a persistence of the RAD52 protein onto chromatin suggesting a possible repair *via* the SSA (**Figure 4B**). Moreover, to confirm the resolution of DSBs, induced by CPT and DHO cotreatment, through the SSA mechanism we analyzed the chromatin loading of the nuclease ERCC1 which act downstream to RAD52 protein (31). As reported in **Figure 4C**, the presence of DHO increases a chromatin loading of ERCC1 in response to CPT treatment. Finally, we performed a GFP reporter assay in HeLa cells stably expressing the coding plasmid hprtSAGFP to monitor the SSA activity in response to DHO treatment (22). Consistently with the previous experiments, the presence of DHO increased the activity of the SSA repair mechanism respect to the vehicle alone (**Figure 4D**).

### 3.4 DHO extract induces an increased sensitivity in different cancer cell lines to CPT treatment

**Figure 5:**
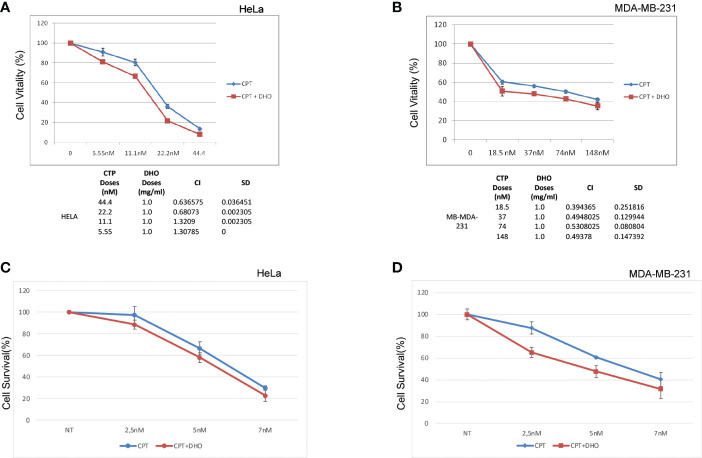
Synergistic effect of DHO-CPT combination on different cancer cell lines **(A)** HeLa cells were pretreated with 1 mg/ml of DHO hydrophilic extract or vehicle (DMSO) followed by different doses of CPT for additional 72 hours and cell viability was analyzed by MTS assay. Results represent the means and SD of three independent experiments, each conducted in triplicate, and are expressed as percentages of cell viability, calculated with respect to the control cells treated with DMSO alone. Combination index was calculated as described in matherial and methods. **(B)** MDA-MB-231 cells were pretreated with 1mg/ml of DHO or vehicle followed by incubation with different doses of CPT for additional 72 hours and cell viability assay was performed as previously. **(C)** HeLa cells were pretreated with DHO at 1mg/ml for one hour followed by treatment with CPT at indicated concentrations; at the end of ten days of incubations, Hela cells were stained with crystal violet. **(D)** MDA-MB-231 cells were incubated with DHO, as described previously, for the cell viabilty assay and cultured for ten days followed by staining with the cristal violet.

We assessed, through MTS assay, the effect of CPT on HELA and MD-MBA-231 cell viability at 72h after treatment (**Figures 5A, B**). The treatment showed a dose-dependent cytotoxic effect. Conversely, our preliminary data show that DHO does not reduce viability of all cell lines tested (data not shown). We examined whether CPT synergizes with DHO in both HELA and MB-MDA-231 cells by MTS assay. Based their approximately IC50 values, previously calculated for CPT, we challenged our cells for 72 h with various doses of CPT in combination with 1mg/ml of DHO. The MTS data obtained were analyzed through the Chou-Talalay method and the CI value of HELA cells indicate synergism for 44.4 nM and 22.2 nM doses for which the CI values revealed is <1 while the CI values of other combination suggests a probably additive effect for HELA cells. Interesting, the data regarding breast cancer cell lines, indeed, show that all doses of CPT combined with 1mg/mg of DHO are synergic, all CI values in breast cancer cell lines are >1 (**Figures 5A, B**). Finally, in order to verify if DHO treatment exerts a long-time effect on cell proliferation, we performed a long-term cell survival assay using DHO and CPT both alone and in combination. Consistently, our data shown that DHO treatment increases the CPT cytotoxic effect in both cancer cell lines (**Figures 5C, D**).

## 4 Discussion

Recently, the National Cancer Institute developed a research program, called the NCI Program for Natural Products Discovery (NPNPD), in order to identify novel natural compounds for cancer therapy. Here, we tested a new tomato genotype, named DHO, as possible agent able to improve the efficacy of standard cancer therapy. We demonstrated the effects of this DHO hydrophilic extract on the RPA32 S4/8 phosphorylation induced by CPT leading an altered RAD52 chromatin loading; moreover, the use of the DHO extract in combination with the CPT treatment induces a marked sensitivity in HeLa cell lines to topoisomerase inhibitor treatment showing a promising mechanism for cancer therapy. We analyzed the possible effect of the hydrophilic DHO extract onto regulation of RPA32S4/8 phosphorylation in response to CPT treatment. RPA complex is one of the principal components of replication and DNA repair machineries (32); in particular, it is involved in the single strand DNA binding reducing the torsional stress (32). In the DNA repair mechanism, RPA plays a central role in the DNA end-resection through its binding to the 3’ tails favoring the resolution of the HR through the single strand-annealing (SSA) or Gene Conversion (GC) (3). The S4/8 phosphorylation of RPA32 occurs when the complex binds to the ssDNA and it is described to be a DNA end resection marker (33–35). So, the modulation of RPA32 phosphorylation, mediated by the DHO extract, could be an indication of increased ssDNA tails and it can influence the resolution of the HR intermediates. Several studies indicated that CPT induces an enhancement of radical oxygen species leading to apoptosis mediated by the mitochondrial membrane modification (36). Here, we demonstrated how the DHO extract was able to modify the DNA damage response induced by CPT treatment, probably by a different mechanism of action compared to UVA response (16). Moreover, it was hypothesized that the protective role of DHO to the UVA induced DNA damage could be dependent on the high amount of AsA present in the hydrophilic extract (16). Conversely, the modulation of the DDR by the DHO extract seems to be not dependent on the Vitamin C, suggesting a possible new molecule involved in the RPA32 regulation. The DNA double strand breaks are repaired by the HR and NHEJ, whose choice depends on the presence of many proteins, as the balance between the BRCA1 and 53BP1, in order to preserve the genome stability (37). DNA end-resection mechanism generates the 3’ single strand tails necessary for the proper HR. Recently, a novel resection dependent c-NHEJ is described but it seems to be a G1 phase pathway (30). Our data shown that the DHO extract doesn’t impair the HR and c-NHEJ suggesting a normal DNA pathway choice in presence of this hydrophilic tomato extract. Once the DNA end-resection occurs, three pathways could compete each other to repair the damaged DNA: GC, alt-EJ and SSA. The GC pathway is essential for the genome stability maintenance given the presence of the sister chromatid for the DNA synthesis mediated repair. By the DR-GFP reporter assay, we demonstrated that DHO extract does not affect the GC conversion rate. The other two pathways, alt-EJ and SSA, are mutagenic DNA repair mechanisms, which are differently regulated: PARP1 mediates the annealing of the microhomologies (38) in conjunction with the Polq, which displaces the RPA from the DNA lesions (39); furthermore, RPA complex inhibits the microhomologies annealing (40). Conversely, RAD52 protein is the main regulator of the SSA favoring the annealing of exposed repeats (7). The dynamics of SSA regulations are not fully understood, but it was described to be dependent on the high resected DNA levels, as demonstrated by the increased RAD52-ssDNA binding in absence of the anti-resection protein 53BP1 (41). Moreover, RAD52 seems to bind preferentially a long stretch of ssDNA (42); we showed an increased RPA32 S4/8 phosphorylation, a marker of DNA end resection, in presence of DHO hydrophilic extract consistently with an enhanced resected DNA; moreover, we showed a marked RAD52 and ERCC1 binding to chromatin fraction of HeLa DHO treated cells, suggesting a possible regulation of SSA. Finally, we demonstrated the potential role of DHO hydrophilic extract to increase the CPT cytotoxicity through the cell vitality assay; this was an interesting phenomenon, supported by the possible role of DHO extract in the modulation of SSA, which is a mutagenic DNA repair mechanism leading to increased cancer cell death. Importantly, the DHO extract alone did not affect the cell viability. In conclusion, this study demonstrated the potential role of the tomato hydrophilic extract in the modulation of DDR induced by Campthotecin treatment leading to a repair through the mutagenic SSA pathway. Moreover, the increased sensitivity in different cancer cell lines to CPT and DHO co-treatment, respect to CPT alone, suggest a possible presence of a molecule into the DHO extract which is able to enhance the efficiency of cancer therapy supporting the further identification of this molecule from the tomato extract. 

## Data availability statement

The raw data supporting the conclusions of this article will be made available by the authors, without undue reservation.

## Author contributions

Conceptualization, LA, AG, ML; methodology, DB, CI, IF, M. MD’Aq, AA, RC, AC, MM, MD’An; tomato extract preparation, MMR, DM, LF, AB, PI; writing—original draft, LA, AG. All authors contributed to the article and approved the submitted version.
